# Impacts of the COVID-19 Pandemic on Family Mental Health in Canada: Findings from a Multi-Round Cross-Sectional Study

**DOI:** 10.3390/ijerph182212080

**Published:** 2021-11-17

**Authors:** Kimberly C. Thomson, Emily Jenkins, Randip Gill, Chris G. Richardson, Monique Gagné Petteni, Corey McAuliffe, Anne M. Gadermann

**Affiliations:** 1 Human Early Learning Partnership, School of Population and Public Health, Faculty of Medicine, University of British Columbia, Vancouver, BC V6T 1Z3, Canada; r.gill@ubc.ca (R.G.); monique.gagne@ubc.ca (M.G.P.); anne.gadermann@ubc.ca (A.M.G.); 2 Centre for Health Evaluation and Outcome Sciences, Providence Health Care Research Institute, British Columbia, Vancouver, BC V6Z 1Y6, Canada; chris.richardson@ubc.ca; 3 School of Nursing, Faculty of Applied Science, University of British Columbia, Vancouver, BC V6T 2B5, Canada; emily.jenkins@ubc.ca (E.J.); corey.mcauliffe@ubc.ca (C.M.); 4 School of Population and Public Health, Faculty of Medicine, University of British Columbia, Vancouver, BC V6T 1Z3, Canada

**Keywords:** COVID-19, mental health, parents, children, families

## Abstract

Pandemic-related disruptions, including school, child care, and workplace closures, financial stressors, and relationship challenges, present unique risks to families’ mental health. We examined the mental health impacts of the coronavirus disease 2019 (COVID-19) pandemic among parents with children <18 years old living at home over three study rounds in May 2020 (*n* = 618), September 2020 (*n* = 804), and January 2021 (*n* = 602). Data were collected using a cross-sectional online survey of adults living in Canada, nationally representative by age, gender, household income, and region. Chi-square tests and logistic regression compared outcomes between parents and the rest of the sample, among parent subgroups, and over time. Parents reported worsened mental health compared with before the pandemic, as well as not coping well, increased alcohol use, increased suicidal thoughts/feelings, worsened mental health among their children, and increases in both negative and positive parent–child interactions. Mental health challenges were more frequently reported among parents with pre-existing mental health conditions, disabilities, and financial/relationship stressors. Increased alcohol use was more frequently reported among younger parents and men. Sustained mental health challenges of parents throughout nearly a year of the pandemic suggest that intervention efforts to support family mental health may not be adequately meeting families’ needs. Addressing family stressors through financial benefit programs and virtual mental health supports should be further explored.

## 1. Introduction

The COVID-19 pandemic has resulted in a substantial burden to population mental health worldwide, with experts calling on mental health professionals and health systems to prepare for long-term and potentially time-lagged increases in mental health demands [1,2]. Population subgroups at greater risk of deteriorated mental health have also been identified, including young people, frontline workers, those who contracted the COVID-19 virus, and people with pre-existing mental health conditions [2,3,4]. The current study sought to explore the self-reported impacts of the pandemic on another population subgroup at potentially heightened mental health risks—parents with children living at home [5,6,7,8,9]. In addition to the anxieties and harms associated with the disease itself, financial and employment stressors, school and child care closures, caregiver burden, and physical distancing from family and friends have created unprecedented disruptions to the lives of families [6,8]. Understanding the pandemic-related stressors facing parents and children is therefore critical to maintaining and strengthening the mental health of families during the pandemic and beyond.

### 1.1. Worsened Family Mental Health in the Context of the COVID-19 Pandemic

Research in Australia has identified that parents with children living at home have reported higher rates of depression, anxiety, and stress, as well as increased alcohol use compared with pre-pandemic levels [10]. The same study identified that younger parents, parents experiencing financial stress, and those with pre-existing mental health conditions reported worse mental health compared with other parents, as well as more strained family relationships [10]. Specifically, pre-existing financial deprivation and COVID-19 stressors were associated with greater severity in parents’ and children’s mental health challenges, parent emotion dysregulation, parenting irritability, domestic conflict, and lower positive expressiveness (e.g., expressing love) with family members [10]. Similarly, predictors of parenting-related exhaustion during the pandemic have included parent gender (women reporting more exhaustion than men), fewer perceived social connections, single-parent status, psychological distress, and lower parental resilience, as well as having a child with special needs, having a large number of children, and having younger children [11].

The pandemic has furthermore significantly impacted children’s mental health. Interviews with families during the pandemic have identified adverse mental health effects among children and adolescents, including feelings of social isolation, depression, anxiety, and increases in behavior problems [12]. Research in Canada has also documented increases in children’s self-reported depression, anxiety, irritability, attention problems, and hyperactivity [13]. Stressors for children include changes within the home environment, including parental stress, loneliness, and disruptions to the school routine among school-aged children [14,15,16,17]. In addition to the immediate impacts on children’s mental health, a further concern for young people is the long-term developmental impacts of chronic stress on their social and emotional health, further emphasizing the need to understand the factors that reduce family stressors [18].

Importantly, the adverse impacts of the pandemic on family mental health are not equally distributed. Disruptions to school routines, for example, have been more likely to impact families experiencing social and structural marginalization who experience compounding stressors, including financial strain and barriers to support their children’s online learning during the pandemic [19]. In turn, higher parenting stress has been associated with elevated child anxiety [20]. Children with chronic health conditions, disabilities, and pre-existing mental health challenges have been particularly impacted by the pandemic due to increased stressors within the home environment, interruptions to routines and care, and health risks of viral exposure [12,17,18].

### 1.2. Impact of the Pandemic on Parent–Child Interactions

Pandemic-related stressors have also impacted parent–child interactions. Increased household chaos, compromised home, school, and work environments, isolation from peers and child care support, and increased worry and uncertainty among both parents and children have created conditions that may challenge the parent–child relationship [21,22]. Higher levels of parenting stress have been associated with increased use of harsh parenting practices [23]. In addition, parents who experienced financial stress [24] or job loss during the pandemic have been found to be at greater risk of child maltreatment [25]. Other stressors on parent–child relationships have included caregiver depression, unmet child care needs, and relationship distress among domestic partners [26].

In contrast, other research highlights that the pandemic has also created opportunities for families to spend more time together, potentially increasing feelings of closeness [21,27]. Since the onset of the pandemic, some families have reported greater structure and positive interactions, such as eating meals together [28]. In addition, maintaining a structured, predictable home environment by adhering to family routines appears to mitigate the adverse effects of the pandemic on children’s mental health [29]. Maintaining good communication, engaging in shared activities, seeking social support, and practicing gratitude have also been identified as factors helping families cope with stress during the pandemic [30].

### 1.3. Current Study

The current study presents findings on the mental health impacts of the COVID-19 pandemic on families in Canada from three rounds of data collection spanning from May 2020 to January 2021. We examined research questions across three objectives: (1) How has the COVID-19 pandemic been associated with the mental health of parents and children at different timepoints during the pandemic, and are there mental health inequities according to social and structural marginalization? (2) How have parent–child interactions changed during the pandemic? (3) What are the factors that support parent and child mental health in the family context? Our analysis was largely exploratory; however, based on the literature, we hypothesized that self-reported mental health outcomes would be worse among parents facing social and structural marginalization, including pre-existing mental health challenges and disabilities, as well as pandemic-related stressors, including financial concerns, relationship challenges, and looking after children while continuing to work.

## 2. Materials and Methods

### 2.1. Sample and Procedure

Data were drawn from a nationally representative multi-round cross-sectional survey study, “Assessing the Impacts of COVID-19 on Mental Health,” a collaboration between the University of British Columbia, the Canadian Mental Health Association (Canada), and the Mental Health Foundation (UK). The current investigation focused on participants who identified as parents/guardians with children <18 years old currently living at home. 

Data were collected using a rapid online survey. Round 1 data were collected on 14–29 May 2020, round 2 data were collected on 14–21 September 2020, and round 3 data were collected 22–28 January 2021. Round 1 corresponded with the first phases of “re-opening” across several Canadian provinces and territories, after approximately 2 months of pandemic-related disruptions, including mandated physical distancing and school/child care and work closures. The 7-day average of new daily cases in Canada was between 1079 and 1247 [31]. Round 2 corresponded with the return to school in many provinces following 2 months of summer holidays that allowed gathering within social bubbles and meeting with others in outdoor spaces. The 7-day average of new daily cases was between 554 and 1833 [31]. Round 3 corresponded with another return to school in many provinces following 2 weeks of winter holidays and the highest yet documented peak in cases. The 7-day average of new daily cases was between 4679 and 4806 [31].

In each study round, surveys were distributed to a randomly selected cross section of the Maru Voice Canada panel (approximately 125,000 adults) maintained by the polling vendor Maru/Matchbox. Selection was stratified based on Canadian national census definitions with adjustments for response propensity, to generate a sample representative by age, gender, household income, and province/territory [32]. Direct emails were sent to panel participants, and targeted sampling was conducted through affiliate community networks to encourage participation from populations that may be harder to reach via the internet [32]. Response-to-invitation ratios were 32% (round 1), 36% (round 2), and 36% (round 3) to yield a sample of approximately 3000 adult participants per round. Subsamples of these respondents identified as parents with children <18 years old living at home in round 1 (*n* = 618), round 2 (*n* = 804), and round 3 (*n* = 602). Within the overall parent subsample, 483 respondents (24%) participated in more than one survey round. There were no differences between respondents who participated in multiple versus one survey round, according to age, disability, or pre-existing mental health conditions. A higher proportion of men participated in multiple survey rounds compared with women.

Consent was provided online before starting the survey. All participants were provided with a small honorarium through Maru/Matchbox to compensate for their time. Ethics approval was provided by the Behavioural Research Ethics Board at the University of British Columbia (H20-01273).

### 2.2. Measures

Survey items were informed by a monitoring survey first commissioned by the Mental Health Foundation in March 2020 (London, UK). The original survey was developed in consultation with people with lived experience of mental health conditions via a citizens’ jury participatory methodology process [33]. To reflect the Canadian context, the research team made modifications to the survey in consultation with collaborators from the Canadian Mental Health Association. Additional survey items were added in round 2 to examine stress related to return to school/child care; as a result, a limited selection of survey items were missing in round 1 and could not be compared across timepoints. A full list of survey items is provided in the Supplementary Materials.

### 2.3. Outcome Variables

Worsened mental health was measured by the question “Compared with before the COVID-19 pandemic and related restrictions in Canada, how would you say your mental health is now?” Responses were dichotomized as worse (slightly or significantly worse now) or not worse (about the same, slightly better, significantly better).

Not coping well was measured by the question “Overall, how well do you think you are coping with stress related to the COVID-19 pandemic?” Responses were dichotomized as not well (not very well or not well at all) or other (fairly well, very well).

Increased alcohol use was measured by asking participants how much their consumption of alcohol has been impacted by the COVID-19 pandemic. Responses were dichotomized as more or not more (less, no change).

Suicidal thoughts/feelings were measured by asking participants if they had experienced suicidal thoughts/feelings as a result of the COVID-19 pandemic in the past 2 weeks. Responses were dichotomized as yes or not yes (no, don’t know).

Children’s worsened mental health was measured by the question “Compared with before the COVID-19 pandemic and related restrictions in Canada, how would you say the mental health of your child/children is now?” Responses were dichotomized as worse (slightly or significantly worse now) or not worse (about the same, slightly better, significantly better, it is affecting my children differently).

Changes to parent–child interactions were measured using eight items adapted from previously developed community survey items related to the COVID-19 pandemic from the University of Michigan [34]. Parents were asked to indicate how their interactions with their children had been impacted by the COVID-19 pandemic. Example parent–child interactions included having quality time, feeling closeness, or using harsh words. Responses were dichotomized as more or not more (less, no change).

### 2.4. Independent Variables

Parent background characteristics and pandemic-related stressors were examined as independent variables. These included parent age, gender, pre-existing mental health condition(s), and disability. Parents were also asked if as a result of the COVID-19 pandemic in the past 2 weeks they had been stressed or worried about financial concerns or relationship challenges or looking after children while continuing to work. Responses were dichotomized as yes or not yes (no, don’t know).

### 2.5. Analyses

To address the first research objective, we compared changes in parent and child mental health associated with the pandemic across the three study rounds. At each round, we conducted chi-square analyses to compare differences in self-reported mental health between parents and the rest of the overall sample (i.e., respondents who were not parents with children <18 years old living at home). Within the parent subsample, we pooled data across the three study rounds and used logistic regression to examine associations between sociodemographic characteristics, stressors, and changes in mental health. To meet the assumptions of independent samples for regression analysis, we included the earliest completed survey if participants had completed multiple survey rounds. Survey round was also included as a covariate in these models to examine trends over time. To address the second research objective, we compared changes in parent–child interactions across each study round. Logistic regression was conducted to examine associations between parent stressors (financial concerns, relationship challenges, and looking after children while continuing to work) and changes in parent–child interactions, adjusting for parent background and study round. To address the third research objective, we calculated frequencies of parent-reported strategies to cope with pandemic-related stressors.

Data were analyzed using SPSS version 26 (SPSS Statistics for Macintosh, Version 26.0., IBM, Armonk, USA). The maximum margin of error for proportions derived from the parent subsample at a 95% level of confidence was ±3.9% in round 1, ±3.5% in round 2, and ±4.0% in round 3. This was a complete case analysis. In chi-square and logistic regression analyses, “don’t know,” “not applicable,” and “prefer not to answer” responses were treated as “not yes.”

## 3. Results

### 3.1. Sample Description

In round 1, 618 of the 3000 respondents identified as parents to a child <18 years old living at home. The average age of the parent subsample was 43.0 years (SD = 9.0 years), and 52.4% identified as women. In round 2, 804 of the 3027 respondents identified as parents, average age of 44.0 (SD = 8.9 years), 54.4% women. In round 3, 602 of the 3034 respondents identified as parents, average age of 43.0 (SD = 8.8 years), 53.5% women. Further sample characteristics are presented in Table 1.

### 3.2. Mental Health of Parents and Children

Table 2 presents the proportions of parents reporting changes in mental health outcomes over the 8-month study period. Chi-square statistics comparing mental health outcomes between parents and the rest of the overall sample (i.e., adults without children living at home) are presented in Supplementary Table S1. The proportion of parents reporting worsened mental health ranged from 44.4% in round 1 to 40.8% in round 2 to 42.2% in round 3 (95% confidence intervals (CIs) provided in Table 2). In round 1, a significantly higher proportion of parents with children at home reported worsened mental health compared with the rest of the overall sample (35.6%). In rounds 2 and 3, the proportion of respondents reporting worsened mental health did not differ between parents and the rest of the overall sample (rest of sample, 38.2% and 39.3% in rounds 2 and 3, respectively).

The proportion of parents who reported not coping well ranged from 16.0% in round 1 to 19.6% in round 2 to 19.0% in round 3. In round 1, there was no difference in the proportion of parents who reported not coping well compared with the rest of the overall sample (13.2%). In rounds 2 and 3, a significantly higher proportion of parents with children at home reported not coping well compared with the rest of the overall sample (12.2% and 13.8%, respectively).

The proportion of parents reporting increased alcohol use ranged from 27.7% in round 1 to 21.9% in round 2 to 22.4% in round 3. Across all three study rounds, a significantly higher proportion of parents with children at home reported increased alcohol use compared with the rest of the overall sample (16.1% in round 1, 14.8% in round 2, and 15.4% in round 3).

The proportion of parents reporting suicidal thoughts/feelings ranged from 8.4% in round 1 to 8.3% in round 2 to 7.9% in round 3. A significantly higher proportion of parents with children at home reported suicidal thoughts/feelings compared with the rest of the sample in round 1 (5.3%) but not in rounds 2 or 3 (rest of sample, 7.4% and 6.1%, respectively).

Within the parent subsample, the proportion of parents reporting worsened mental health for one or more of their children ranged from 24.8% in round 1 to 25.7% in round 2 to 30.9% in round 3.

### 3.3. Pandemic-Related Stressors

**Figure 1:**
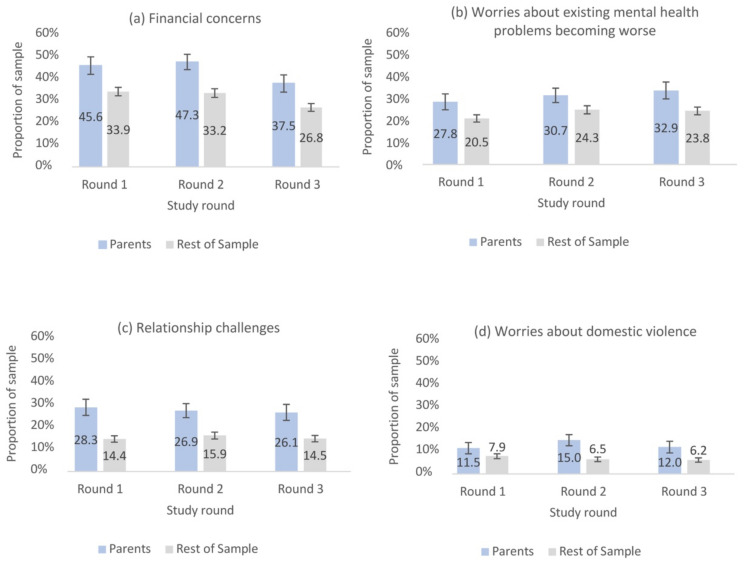
Proportion of parents compared with the rest of the sample reporting pandemic-related stressors, by study round (error bars represent 95% confidence intervals); (**a**) Financial concerns, (**b**) Worries about existing mental health problems becoming worse, (**c**) Relationship challenges, (**d**) Worries about domestic violence.

Figure 1 presents the proportion of stressors reported by parents and the rest of the sample over the study period. Financial concerns were frequently reported by parents, with 45.6% of parents reporting being stressed/worried about financial concerns in round 1, 47.3% in round 2, and 37.5% in round 3. A significantly higher proportion of parents compared with the rest of the overall sample reported this stressor in all three study rounds (rest of sample, 33.9% in round 1, 33.2% in round 2, and 26.8% in round 3, χ^2^ (1, *n* = 3000–3034) = 27.3–29.1, *p* < 0.001).

Another frequently reported stressor was worrying about existing mental health problems becoming worse, with 27.8% of parents reporting this in round 1, 30.7% in round 2, and 32.9% in round 3. A significantly higher proportion of parents compared with the rest of the overall sample reported this stressor in all three study rounds (rest of sample, 20.5% in round 1, 24.3% round 2, and 23.8% round 3, χ^2^ (1, *n* = 3000–3034) = 12.7–20.9, *p* < 0.001).

Parents reported being stressed/worried about experiencing relationship challenges with their partner, with 28.3% reporting this stressor in round 1, to 26.9% in round 2, and 26.1% in round 3. A significantly higher proportion of parents compared with the rest of the overall sample reported this stressor in all three study rounds (rest of sample, 14.4% in round 1, 15.9% in round 2, and 14.5% in round 3, χ^2^ (1, *n* = 3000–3034) = 46.1–67.0, *p* < 0.001).

Finally, 11.5% of parents reported being stressed/worried about being safe from physical or emotional domestic violence in the past 2 weeks in round 1, 15.0% in round 2, and 12.0% in round 3. Again, a significantly higher proportion of parents compared with the rest of the overall sample reported this stressor in all three study rounds (rest of sample, 7.9% in round 1, 6.5% in round 2, and 6.2% in round 3, χ^2^ (1, *n* = 3000–3034) = 8.0–53.6, *p* < 0.01).

**Figure 2:**
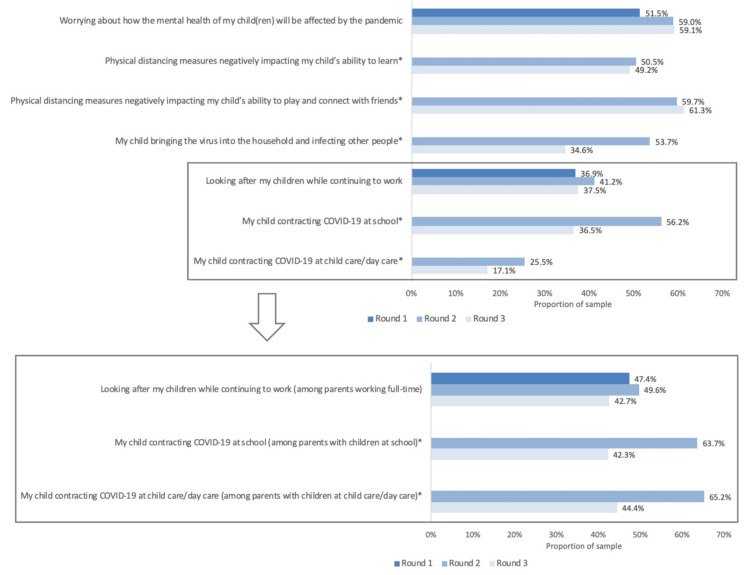
Parent-reported stressors in the past 2 weeks as a result of the COVID-19 pandemic, for the overall parent sample. Inset: Parent-reported stressors among subgroups of parents. * Survey item not asked in round 1.

Parents with children <18 years old at home also faced unique stressors related to parenting during a pandemic (Figure 2). Across the study period, 51.5% to 59.1% of parents reported worrying about the mental health of their children. In the overall parent sample, 36.9% to 41.2% of parents across the study period reported being stressed/worried about looking after children while continuing to work. When the sample was restricted to parents who were working full-time, this proportion increased to 42.7% to 49.6%. In round 2, when many schools were reopening, 56.2% of parents overall reported worrying about their child contracting the virus at school (63.7% when restricted to parents with a child attending school). A further 25.5% of parents overall worried about their children contracting the virus at child care/day care (65.2% when restricted to parents with a child attending child care/day care).

### 3.4. Associations between Parent Sociodemographic Factors, Stressors, and Changes in Mental Health

Table 3 presents associations between the five pandemic-related mental health outcomes (parent self-reported worsened mental health, not coping well, increased alcohol use, suicidal thoughts/feelings, child worsened mental health) and parent background as well as pandemic-related stressors.

Logistic regression analyses identified that over the study period, after adjusting for covariates, parents aged <35 compared with older parents had higher odds of increased alcohol use related to the pandemic but lower odds of perceived worsened mental health of their children. Women compared with men had lower odds of increased alcohol use. Parents with pre-existing mental health conditions compared with those without had two to three times the odds of worsened mental health, not coping well, suicidal thoughts/feelings, and higher odds of perceived worsened mental health of their children. Parents with disabilities compared with those without had higher odds of not coping well.

Parents who were stressed/worried about financial concerns compared with those without this stressor had higher odds of worsened mental health, not coping well, suicidal thoughts/feelings, and perceived worsened mental health of their children. Likewise, parents who were stressed/worried about experiencing relationship challenges with their partner compared with those without this stressor had higher odds of worsened mental health, not coping well, suicidal thoughts/feelings, and perceived worsened mental health of their children. Parents who were stressed/worried about looking after children while continuing to work compared with those without this stressor had higher odds of worsened mental health, increased alcohol use, and perceived worsened mental health of their children. After adjusting for parent background and pandemic-related stressors, parents in round 2 had lower odds of reporting increased alcohol use related to the pandemic compared with round 1.

### 3.5. Changes in Parent–Child Interactions Associated with the Pandemic

Overall, in the context of the COVID-19 pandemic, parents reported increased negative interactions with their children, including conflicts (22.2% in round 1, 18.3% in round 2, 21.6% in round 3), yelling/shouting (16.7% in round 1, 14.3% in round 2, 14.0% in round 3), disciplining (16.0% in round 1, 12.7% in round 2, 12.5% in round 3), and using harsh words (10.7% in round 1, 11.3% in round 2, 9.5% in round 3).

Parents also reported that the pandemic had contributed opportunities for increased positive interactions with their children, including having more quality time (65.4% in round 1, 58.2% in round 2, 56.3% in round 3), feeling closeness (49.7% in round 1, 46.4% in round 2, 47.2% in round 3), showing love or affection to their children (44.5% in round 1, 45.1% in round 2, 41.7% in round 3), and observing increased resilience (strength and perseverance) in their children (38.2% in round 1, 34.8% in round 2, 37.4% in round 3).

Parent-reported stressors were associated with higher odds of both negative and positive interactions with their children (Table 4). After adjusting for study round and parent background (age, gender, pre-existing mental health condition, disability), parents who were stressed/worried about financial concerns had significantly higher odds of nearly all measured negative interactions (increased disciplining, conflicts, harsh words), as well as all measured positive interactions with their children (increased quality time, feeling closeness, showing love or affection, observing resilience). In the adjusted model, parents who were stressed/worried about relationship challenges had significantly higher odds of all measured negative interactions with their children and higher odds of showing love and affection. In the adjusted model, parents who were stressed/worried about looking after children while continuing to work had higher odds of all measured negative and positive interactions with their children.

### 3.6. Supports and Coping throughout the Pandemic

**Figure 3:**
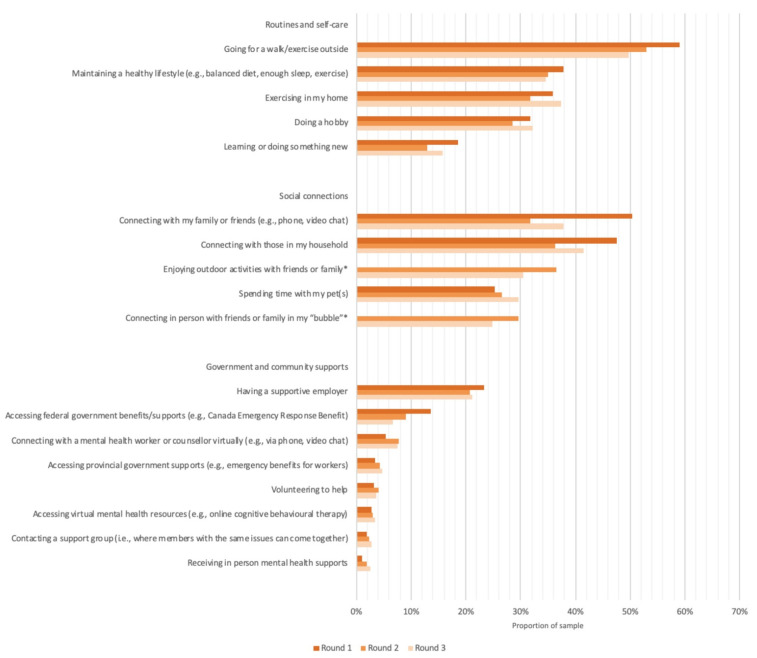
Parent-identified supports for coping with stress related to the COVID-19 pandemic in the past 2 weeks. * Survey item not asked in round 1.

Figure 3 presents sources of support identified by parents that had helped them cope with stress related to the COVID-19 pandemic in the past 2 weeks. Across the study period, the most frequently identified strategies that parents reported included going for a walk/exercise (59.1% in round 1, 53.0% in round 2, 49.7% in round 3), connecting with family and friends via phone and video chat (50.5% in round 1, 31.7% in round 2, 37.9% in round 3), connecting with those in their household (47.6% in round 1, 36.3% in round 2, 41.5% in round 3), and maintaining a healthy lifestyle (37.9% in round 1, 35.1% in round 2, 34.6% in round 3).

There was an indication that connecting with friends and family virtually via phone and video chat was more frequently reported in round 1 (50.5%) compared with round 2 (31.7%) or round 3 (37.9%), whereas connecting in person with friends and family “in my bubble” and enjoying outdoor activities with friends and family were more frequently reported in round 2 (29.6% and 36.4%, respectively) and round 3 (24.8% and 30.6%, respectively). Accessing federal government benefits and supports was more frequently reported in round 1 (13.6%) compared with round 2 (9.1%) or round 3 (6.6%), whereas accessing provincial government supports (e.g., emergency benefits for workers) was less frequently reported in round 1 (3.4%) compared with round 2 (4.4%) or round 3 (4.7%).

By round 3, 60.4% of parents reported experiencing mental health challenges at any point during the pandemic. Of those reporting challenges, 17.9% reported accessing virtual mental health services. The most commonly reported reasons for not accessing virtual mental health services were not feeling in need of help (46.1%), not knowing supports were available (19.1%), and preferring in person healthcare supports (18.8%).

## 4. Discussion

This study identified that cross-sectionally, over the first year of the COVID-19 pandemic, parents with children <18 years old living at home consistently reported worsened mental health, not coping well, increased alcohol use, suicidal thoughts/feelings, and perceived worsened mental health among their children. Across three study rounds, a higher proportion of parents reported mental health challenges during the pandemic compared with respondents who did not have children <18 years old living at home. Specifically, a significantly higher proportion of parents compared with the rest of the sample reported worsened mental health in round 1, not coping well in rounds 2 and 3, increased alcohol use across all three study rounds, and suicidal thoughts/feelings in round 1. There were also significant differences in mental health among parents depending on parent background, with greater reported mental health challenges on one or more outcomes for parents <35 years old, men, parents with pre-existing mental health conditions, and parents with disabilities, as well as among parents with financial concerns, relationship challenges, and stress related to looking after children while continuing to work.

These results align with previous parent-report research suggesting that parents with children at home have been a population at heightened risk of worsened mental health outcomes during the pandemic [6,10,15]. As other studies have identified, a confluence of stressors on families, including economic pressures, balancing multiple demands and disruptions, and increased relational stress, has been associated with poorer mental health and burnout [10,15,35,36]. The current study also identified social disparities in mental health outcomes that concur with past research [15,37]. For example, pandemic-related deteriorations in mental health were elevated among younger parents, for whom a higher proportion reported more alcohol use and suicidal thoughts/feelings compared with other parents. Other Canadian research has similarly identified age gradients in pandemic-related mental health outcomes, with younger adults reporting more anxiety, stress, and depression than older adults [37]. As hypothesized, parents with pre-existing mental health conditions and disabilities and those experiencing financial stress, relationship challenges, and stress related to looking after children while continuing to work also had significantly higher odds of adverse mental health outcomes due to the COVID-19 pandemic, consistent with previous research [10,15].

Overall, we found minimal changes in parents’ self-reported worsened mental health during the first several months of the pandemic and stable inequities in mental health outcomes across the three study rounds. Despite changes in pandemic-related restrictions and supports and services over the study period, after adjusting for parent characteristics and pandemic-related stressors, there were no significant changes in the proportion of parents reporting worsened mental health, not coping well, suicidal thoughts/feelings, or perceived worsened mental health of their children. The only change across study rounds in the adjusted analyses was that increased alcohol use was reported by more parents in round 1 compared with round 2 (but not round 3).

This study also identified several changes in parent–child interactions. Overall, during the study period, parents reported increases in both negative and positive interactions with their children, including increased conflicts and increased closeness and showing affection. This finding is consistent with research in the United States that found parents reported a high level of closeness with their children during the pandemic, as well as increased conflicts, discipline, and harsh words [34]. It contrasts with research in Australia that found decreases in family positive expressiveness during the pandemic [10].

Adjusting for potential confounders, parents who reported stress/worries about financial concerns had higher odds of increased negative and positive interactions with children compared with parents without this stressor. Parents reporting stress/worries about relationship concerns had higher odds of negative interactions with children and higher odds of showing love and affection. Parents who reported stress/worries about looking after children while continuing to work had higher odds of both negative and positive interactions with their children. These associations between stressors and increased negative interactions align with previous research finding that parents who reported greater household chaos and pandemic-related difficulties, including navigating shared spaces, experiencing relationship challenges, and balancing work and family life, also reported increased stress in their relationships with children [22]. The associations between stressors and increased positive interactions also supports earlier suggestions that increased time with family members might create conditions for increases in both negative and positive interactions [27].

Finally, parents in this study identified several strategies and supports that had helped them cope with stress during the pandemic. Across the study period, the most frequently reported strategies included exercise and connecting with friends and family. Coinciding with re-openings for in person visiting in rounds 2 and 3 in many provinces, connecting with friends and family virtually via phone and video chat was less frequently reported in round 2 (31.7%) and round 3 (37.9%) compared with round 1 (50.5%), whereas connecting in person with friends and family in “my bubble” and enjoying outdoor activities with friends and family were more frequently reported in round 2 (29.6% and 36.4%, respectively) and round 3 (24.8% and 30.6%, respectively). Other research has similarly identified the maintenance of family routines, adaptability, and family cohesion as factors of family resilience during the pandemic [30]. Children’s perceived connectedness to caregivers has also been found to predict better mental health outcomes for children during the pandemic [38].

Corresponding with a transition from the Canada Emergency Response Benefit program in October 2020 [39], accessing federal government benefits and supports was less frequently reported in round 2 (9.1%) and round 3 (6.6%) compared with round 1 (13.6%), whereas accessing provincial government supports (e.g., emergency benefits for workers) was more frequently reported in round 2 (4.4%) and round 3 (4.7%) compared with round 1 (3.4%). These trends indicate that improvements are needed to ensure that parents know that these supports are available and that supports are made more easily accessible.

Accessing virtual mental health supports was infrequently reported throughout the study period, with only 3.5% of the total parent sample reporting accessing these supports as a way to cope with stress from the pandemic in the past 2 weeks. This is consistent with related research using the same survey data that found only 2.0% of the general population in Canada had accessed virtual health services in May 2020 [40]. The reasons for not accessing virtual mental health services were also comparable to the barriers identified in previous research, including not feeling in need of help, not knowing supports were available, not having time or energy, preferring in person healthcare supports, and not believing it would help [40,41]. However, greater uptake was observed among parents reporting mental health challenges (17.9%), suggesting that virtual mental health services may be an underutilized but nonetheless important avenue to meet a growing demand for mental health supports as the pandemic and its social and economic repercussions continue. The mental health impacts of the pandemic are expected to endure beyond the physical health impacts, necessitating sustained investments in mental health services and systems [1,2,4,42]. Relevant to health professionals and service providers, this study identifies parents as a priority population subgroup reporting significant mental health challenges during the pandemic. Incorporating questions about mental health may be a particularly important practice for general practitioners, who may be the first point of contact for many parents who have not previously engaged with mental health services.

### Strengths and Limitations

There are key study strengths and limitations to highlight for the reader. This study facilitated data collection in the midst of the COVID-19 pandemic with a resulting sample that was large and nationally representative according to age, gender, household income, and province/territory. This sample enabled us to gain a better understanding of the disparities in mental health experienced specifically by parents and families during the COVID-19 pandemic. Although the study was designed to capture population subgroups of parents from diverse backgrounds, the sample sizes of certain subgroups were too small to allow us to conduct meaningful subgroup analysis (e.g., parents identifying as Lesbian, Gay, Bisexual, Trans, Two-Spirit, Queer, and additional identities (LGBT2Q+) or Indigenous parents). We employed sampling methodologies that were designed to maximize representativeness and minimize selection bias (e.g., oversampling, community partner engagement), but we note that there remains the possibility that survey respondents differed from the general population on key factors, such as their mental health, education and occupational status, and family circumstances, potentially impacting the estimates presented here. It is also important to remind the reader that this was a multi-round cross-sectional study, and we did not measure trends in mental health among the same participants over time. Because exposures and outcomes were measured simultaneously at each round, the findings need to be interpreted as associations rather than causation. In the logistic regression analyses, we furthermore excluded responses from 24% of participants who overlapped across study rounds, which may have opened the potential for sampling bias. However, we adjusted for select parent characteristics to address this limitation.

This study was furthermore designed to measure self-reported mental health associated with the COVID-19 pandemic at the community level rather than assess the prevalence of diagnostic mental health outcomes. We did not include clinical mental health assessments, and these measurement differences may limit comparability with other research. Items were adapted from previous surveys where possible, and new items were developed with input from people with lived experience of mental health conditions, researchers, and mental health service providers. However, a trade-off of this approach was that survey items designed for this study were not validated and again may not be comparable with other research. Future research examining clinical indicators of changes in mental health using standard screening measures would be beneficial to health service providers and policy makers. We also did not have baseline measures of mental health prior to the pandemic, although we were able to control for self-reported pre-existing mental health conditions. Furthermore, the results of this study are specific to the Canadian context and may not reflect the impact of the pandemic on family mental health in different settings. The current results also average the family experience within Canada and do not account for geographical variations, including differences in pandemic-related restrictions over the study period by province. Additionally, the parent-report design of this study meant that we could only assess changes to children’s mental health from the perspectives of parents. Assessing the impact of the pandemic from the perspectives of children and youth themselves is an important future research direction.

## 5. Conclusions

Results from this study support the suggestion that changes and disruptions related to the COVID-19 pandemic have presented unique risks to families’ perceived mental health [15,43]. Regarding our first research objective, we found that deteriorations in families’ mental health remained relatively stable over the first several months of the pandemic; parents reported similar levels of worsened mental health, not coping well, increased alcohol use, suicidal thoughts/feelings, and worsened mental health among their children across study rounds. This study also identified mental health inequities faced by subgroups of parents, most notably parents with pre-existing mental health conditions and those experiencing financial and/or relational stressors. These results suggest that current intervention efforts to support family mental health may be not adequately meeting families’ needs or closing gaps in social disparities. Regarding our second objective, we found that despite these sustained stressors and impacts on mental health, many parents reported increases in both negative and positive interactions with their children. This result supports the notion that the pandemic, for some families, may have also been a time to strengthen relationships [27]. Finally, regarding our third objective, maintaining family routines and social connections with family and friends in and outside the home were consistently reported by parents as supports that had helped them cope with stress due to the pandemic. Future research should continue to investigate how services including financial benefits and virtual mental health supports can be improved for families to further optimize these supports.

## Figures and Tables

**Table 1:** Sociodemographic characteristics of the parent sample, by survey round.

	Round 1 May 2020 (*n* = 618)		Round 2 September 2020 (*n* = 804)		Round 3 January 2021 (*n* = 602)	
	*n*	%	*n*	%	*n*	%
Gender						
Men	294	47.6	361	44.9	277	46.0
Women	324	52.4	437	54.4	322	53.5
Nonbinary	0	0.0	3	0.4	2	0.3
Age						
18–34 years	130	21.0	107	13.3	105	17.4
35–54 years	449	72.7	606	75.4	451	74.9
≥55 years	39	6.3	91	11.3	46	7.6
Province						
Alberta	86	13.9	99	12.3	80	13.3
British Columbia/Territories	81	13.1	99	12.3	77	12.8
Manitoba/Saskatchewan	49	7.9	48	6.0	39	6.5
Atlantic Provinces	43	7.0	148	18.4	38	6.3
Ontario	243	39.3	288	35.8	247	41.0
Quebec	116	18.8	122	15.2	119	19.8
Rural/Urban						
Urban	531	85.9	641	79.7	468	77.7
Rural	87	14.1	163	20.3	134	22.3
Education						
High school or less	62	10.0	96	11.9	61	10.1
Some college or university	81	13.1	117	14.6	71	11.8
College or university graduate	475	76.9	591	73.5	470	78.1
Marital status						
Single, never married	39	6.3	55	6.8	31	5.1
Married or partnered	517	83.7	673	83.7	527	87.5
Separated, divorced, widowed	62	10.0	76	9.5	44	7.3
Income						
<$50,000	108	17.5	112	14.2	80	13.7
$50,000 to <$100,000	197	31.9	293	37.3	193	33.0
≥$100,000	313	50.6	381	48.5	312	53.3
LGBT2Q+ ^a^						
Yes/unsure	28	4.5	47	5.8	21	3.5
Pre-existing mental health condition						
Yes	111	18.0	140	17.4	94	15.6
Disability						
Yes	45	7.3	76	9.5	36	6.0
Visible minority						
Yes	118	19.1	161	20.0	128	21.3
Household living situation (check all that apply)						
Living with spouse/partner	500	80.9	660	82.1	511	84.9
Living with other adults	47	4.1	51	3.4	38	3.3
Living with grandchildren	11	1.8	18	2.2	15	2.5
Age of children at home (check all that apply)						
<4 years	183	29.6	173	21.5	146	24.3
5–11 years	292	47.2	424	52.7	299	49.7
12–17 years	309	50.0	427	53.1	299	49.7
≥18 years	70	11.3	119	14.8	78	13.0
Child siblings at home						
Yes	379	61.3	546	67.9	377	62.6

^a^ Lesbian, Gay, Bisexual, Trans, Two-Spirit, Queer, and additional identities.

**Table 2:** Proportion of parents reporting mental health outcomes, by survey round.

	Round 1 (May 2020) *n* = 618 ^a^				Round 2 (October 2020) *n* = 804 ^a^				Round 3 (January 2021) *n* = 602 ^a^			
	*n*	%	95% CI		*n*	%	95% CI		*n*	%	95% CI	
Parent Worsened Mental Health	274	44.4%	40.4%	48.4%	327	40.8%	37.4%	44.3%	254	42.2%	38.2%	46.3%
Not Coping Well	97	16.0%	13.2%	19.2%	154	19.6%	16.9%	22.6%	110	19.0%	15.9%	22.4%
Increased Alcohol Use	171	27.7%	24.2%	31.4%	176	21.9%	19.1%	24.9%	135	22.4%	19.2%	26.0%
Suicidal Thoughts/Feelings	51	8.4%	6.3%	10.9%	66	8.3%	6.5%	10.4%	47	7.9%	5.9%	10.4%
Child Worsened Mental Health	153	24.8%	21.4%	28.4%	207	25.7%	22.8%	28.9%	186	30.9%	27.2%	34.8%

^a^ Denominators varied slightly due to missing data.

**Table 3:** Odds ratios of worsened mental health since the onset of the COVID-19 pandemic by parent sociodemographic factors, stressors, and survey round (*n* = 1541) ^a^.

	Parent Worsened Mental Health			Parent Not Coping Well			Parent Increased Alcohol Use			Parent Suicidal Thoughts/ Feelings			Child Worsened Mental Health		
	OR ^b^	95% CI ^c^		OR	95% CI		OR	95% CI		OR	95% CI		OR	95% CI	
**Parent background**															
Age < 35	1.15	0.86	1.56	1.35	0.93	1.95	**1.51**	1.10	2.07	1.43	0.87	2.34	**0.71**	0.50	0.99
Women	1.13	0.90	1.41	1.07	0.80	1.44	**0.76**	0.59	0.97	0.74	0.49	1.11	1.01	0.79	1.29
Pre-existing mental health condition	**2.21**	1.65	2.98	**2.65**	1.90	3.70	1.21	0.88	1.68	**3.51**	2.28	5.41	**1.43**	1.05	1.95
Disability	1.31	0.85	2.00	**1.60**	1.01	2.55	1.06	0.66	1.70	1.54	0.85	2.83	1.14	0.73	1.77
**Parent stressors**															
Financial concerns	**1.58**	1.26	1.97	**1.70**	1.26	2.30	0.98	0.76	1.26	**1.90**	1.24	2.92	**1.30**	1.02	1.67
Experiencing relationship challenges with my partner	**2.15**	1.67	2.76	**2.60**	1.91	3.53	1.24	0.94	1.65	**1.63**	1.06	2.49	**1.58**	1.21	2.06
Looking after children while continuing to work	**1.59**	1.27	1.99	1.04	0.77	1.40	**1.86**	1.45	2.40	1.36	0.90	2.05	**1.52**	1.19	1.95
**Survey round**															
Survey round 1 (reference)															
Survey round 2	0.84	0.66	1.06	1.29	0.94	1.77	**0.68**	0.52	0.88	0.95	0.62	1.46	0.96	0.74	1.25
Survey round 3	1.12	0.81	1.53	1.12	0.72	1.73	0.77	0.53	1.10	0.72	0.38	1.39	1.39	0.99	1.95

All variables entered simultaneously, adjusting for each other. ^a^ Denominators varied slightly due to missing data. For participants who completed multiple survey rounds, only the earliest completed survey was included. ^b^ Odds ratio. ^c^ Confidence Interval. Bold indicates statistically significant associations.

**Table 4:** Changes in parent–child interactions associated with parent stressors due to the COVID-19 pandemic.

	**Changes in Negative Parent–Child Interactions**											
**Parent Stressors**	**Disciplining My Child(ren)**			**Conflicts with My Child(ren)**			**Using Harsh Words with My Child(ren)**			**Yelling/Shouting at My Child(ren)**		
	**OR**	**95% CI**		**OR**	**95% CI**		**OR**	**95% CI**		**OR**	**95% CI**	
Financial concerns	**1.67**	1.24	2.27	**1.48**	1.15	1.91	**1.69**	1.20	2.39	1.20	0.90	1.61
Experiencing relationship challenges with my partner	**2.63**	1.93	3.57	**2.48**	1.90	3.24	**3.08**	2.18	4.34	**2.75**	2.04	3.71
Looking after children while continuing to work	**3.35**	2.46	4.57	**2.21**	1.71	2.86	**2.19**	1.56	3.08	**2.68**	2.00	3.60
	**Changes in Positive Parent–Child Interactions**											
	**Having More** **Quality Time with My Child(ren)**			**Feeling Closeness with My Child(ren)**			**Showing Love or** **Affection to My Child(ren)**			**Observing** **Resilience (Strength and** **Perseverance)** **in My Child(ren)**		
	**OR**	**95% CI**		**OR**	**95% CI**		**OR**	**95% CI**		**OR**	**95% CI**	
Financial concerns	**1.65**	1.33	2.04	**1.52**	1.24	1.87	**1.71**	1.39	2.10	**1.38**	1.11	1.71
Experiencing relationship challenges with my partner	1.15	0.90	1.46	1.08	0.86	1.36	**1.46**	1.16	1.84	1.20	0.95	1.53
Looking after children while continuing to work	**2.28**	1.82	2.86	**2.07**	1.67	2.55	**2.09**	1.69	2.58	**2.03**	1.64	2.53

Each stressor adjusted for parent age, gender, pre-existing mental health condition, disability, and study round. Bold indicates statistically significant associations.

## Data Availability

The datasets used and/or analyzed during the current study are available from the corresponding author on reasonable request.

## Supplementary Materials

The following are available online at https://www.mdpi.com/article/10.3390/ijerph182212080/s1: Full list of survey items and Supplementary Table S1. Comparison between parents and the overall rest of the sample (adults without children living at home) reporting mental health outcomes, by survey round.
