# Supplementary material for: Impacts of the COVID-19 Pandemic on Family Mental Health in Canada: Findings from a Multi-Round Cross-Sectional Study
Source: Int J Environ Res Public Health. 2021 Nov 17;18(22):12080. doi: 10.3390/ijerph182212080 (PMC8623196; doi:10.3390/ijerph182212080)
Supplement: Supplementary file 1 [file ijerph-18-12080-s001.zip › ijerph-1401628-supplementary.pdf]

Supplementary Material

Survey Items

Assessing the mental health impacts of COVID-19: A national survey study

The 2019 novel coronavirus (2019-nCoV), otherwise known as COVID-19, is an infectious disease that has resulted in a global pandemic. Throughout this questionnaire, we will refer to the disease as COVID-19.

For the following questions, we would like you to think about yourself, members of your household, or other family members who have been affected by the COVID-19 virus or response.

We have provided you with a "Prefer not to answer" option, which you can select if you do not wish to share your experiences on a particular question.

SECTION 1: INTRODUCTION

Age demographics What's your age?

Geographic region In which province or territory do you currently live?
1. Alberta
2. British Columbia
3. Manitoba
4. New Brunswick
5. Newfoundland and Labrador
6. Northwest Territories
7. Nova Scotia
8. Nunavut
9. Ontario
10. Prince Edward Island
11. Quebec
12. Saskatchewan
13. Yukon

Rural Urban Do you live in an urban or rural environment?
1. Urban
2. Rural

Education Please select the highest level of education you have completed.
1. Elementary/grade school
2. Some high school
3. High school graduate
4. Some college / technical school
5. Completed college / technical school
6. Some university
7. University undergraduate degree
8. Some post-graduate school
9. Post-graduate degree

Marital Status What is your marital status?
1. Single, never married
2. Common law
3. Civil partnership
4. Married
5. Separated
6. Divorced
7. Widowed
8. Engaged

Income Please indicate the category which best describes your total household annual income before taxes.
1. Less than $25,000
2. $25,000 to less than $35,000
3. $35,000 to less than $50,000
4. $50,000 to less than $75,000
5. $75,000 to less than $100,000

6. $100,000 to less than $125,000
7. $125,000 to less than $150,000
8. $150,000 to less than $250,000
9. $250,000 to less than $500,000
10. $500,000 or more
11. Don’t know/prefer not to say

SECTION 2: COVID QUESTIONS

Employment Status The COVID-19 pandemic has had substantial impacts on employment. Which of the following best describes your current employment status? (Please select all that apply)

1. Working full time (30 or more hours per week)
2. Working part time (fewer than 30 hours per week)
3. Full time student (e.g. school, college, university, job training)
4. Part time student (e.g. school, college, university, job training)
5. Not working (e.g. parental leave, disability, medical leave, etc.)
6. Not working due to personal preference because of COVID-19
7. Volunteer (unpaid)
8. Retired
9. Unemployed (due to COVID-19)
10. Unemployed (prior to COVID-19)
11. Underemployed (loss of hours due to COVID)
12. Other
13. Prefer not to answer

[If currently working] Essential Service Workers The job that I am currently working in has been deemed as an essential service during the COVID-19 pandemic (i.e., your position is identified by your provincial government as an essential service and you have continued to work in your role throughout the pandemic).

1. Yes
2. No
3. Prefer not to answer

[If yes to essential service worker] Essential Service Worker Category Please select the category that BEST describes your essential service role:

1. Health and health services
2. Law enforcement, public safety, first responder
3. Vulnerable population service provider (e.g., community outreach, substance use and addiction services)
4. Education (e.g., teacher, early childhood educator)
5. Food and agriculture service provider (farming, food processing, grocery, hardware)
6. Transportation
7. Industry and manufacturing
8. Communications and information technology
9. Financial institutions
10. Other
11. Prefer not to answer

Self Mental Health In general, would you say your mental health is:

1. Excellent
2. Very good
3. Good
4. Fair
5. Poor

Mental Health Compared to before the COVID-19 pandemic and related restrictions in Canada, how would you say your mental health is now?

1. Significantly better now
2. Slightly better now
3. About the same
4. Slightly worse now
5. Significantly worse now
6. Prefer not to answer

COVID-19 Disease Which of the following applies to how you have been affected by COVID-19 at any point during the pandemic? (Please select all that apply)

1. I have been tested for COVID-19 and had a positive result
2. I have been tested for COVID-19 and had a negative result
3. Someone else in my household has tested positive for COVID-19
4. Someone else in my household has tested negative for COVID-19
5. A family member/loved one living at a different address has tested positive for COVID-19
6. I have self-isolated with symptoms of COVID-19
7. My household has self-isolated because someone else in my household had symptoms of COVID-19
8. My household has self-isolated due to contact with someone else who had symptoms of COVID-19
9. My household has self-isolated due to recent travel
10. A family member/loved one living at a different address has self-isolated with symptoms of COVID-19
11. As part of my work I have worked directly with individuals who have tested positive for COVID-19
12. I have been hospitalized due to COVID-19
13. Someone in my household has been hospitalized due to COVID-19
14. A family member/loved one living at a different address has been hospitalized due to COVID-19
15. A family member/loved one is living at a long-term care facility that had cases of COVID-19
16. Someone in my household has died due to COVID-19
17. A family member/loved one living at a different address has died due to COVID-19
18. None of these
19. Don't know
20. Prefer not to answer

Emotional Responses Which of the following emotions have you felt as a result of the COVID-19 pandemic in the past 2 weeks? (Please select all that apply)

1. Afraid
2. Panicked
3. Anxious or worried
4. Empathetic

5. Indifferent
6. Hopeful
7. Hopeless
8. Ashamed
9. Guilty
10. Lonely or isolated
11. Unprepared
12. Sad
13. Angry
14. Stressed
15. Bored
16. Inspired
17. Depressed
18. Calm
19. Comfortable
20. Content
21. Secure
22. None of these
23. Don't know
24. Prefer not to answer

Stressors Have you been stressed or worried about any of the following as a result of the COVID-19 pandemic in the past 2 weeks? (Please select one option on each row)

1. Financial concerns (e.g. going into debt, ability to pay bills, long-term economic impacts, etc.)
2. Being unable to access government benefit payments, not being eligible for benefits, or losing benefits
3. Losing my job / loss of my job
4. Being able to cope with uncertainty (e.g. not knowing what will happen)
5. Becoming ill with the virus
6. Having no-one to care for me, as a result of becoming ill with the virus
7. Not being able to care for friends and family as a result of becoming ill
8. Not being able to care for friends and family due to physical distancing
9. Passing COVID-19 on to someone else if I became infected
10. Experiencing discrimination if I contract COVID-19
11. Being vulnerable because of an existing medical condition, age, etc.
12. Being separated from friends and family
13. Being able to cope with physical/social distancing (including concerns when needing to leave my residence for groceries, exercise, health care, etc.)
14. Having enough food to meet my household's basic needs
15. My education or career training has been or will be interrupted
16. Looking after my children while continuing to work
17. Making my existing mental health problems worse
18. Worrying about how the mental health of my child(ren) will be affected by the pandemic
19. My child contracting COVID-19 at childcare/daycare
20. My child contracting COVID-19 at school
21. My child bringing the virus into the household and infecting other people
22. Physical distancing measures negatively impacting my child's ability to learn
23. My child having low motivation and productivity while homeschooling
24. Being able to provide educational support at home while homeschooling my child(ren)
25. Ability of my child to learn at home while homeschooling

26. Physical distancing measures negatively impacting my child's ability to play and connect with friends
27. Contracting the virus in my work setting
28. Being able to access a vaccine when developed
29. The safety or effectiveness of a COVID-19 vaccine when developed
30. Worrying about the impacts of a "second wave" of COVID-19
31. Worrying about the long term mental health impacts resulting from the stress of the pandemic
32. Experiencing relationship challenges with my partner
33. Being safe from physical or emotional domestic violence
34. Fear of getting severely sick or dying
35. Fear of a family member/loved one getting severely sick or dying

    1. Yes
    2. No
    3. Don't know /Not applicable/ Prefer not to say

Sleep During the past 2 weeks, how would you rate your sleep quality overall?
1. Very good
2. Fairly good
3. Fairly bad
4. Very bad

Food Security 1 You and other household members worried that food would run out before you got money to buy more. Was that often true, sometimes true, or never true in the last 12 months?
1. Often true
2. Sometimes true
3. Never true
4. Don't know/prefer not to answer

Food Security 2 Since the onset of the COVID-19 pandemic and related restrictions in Canada, have you or any members of your household accessed food-based community programs to get food? (please select all that apply)
1. Food Bank
2. Soup Kitchens/Free Meal programs
3. Meal or food programs from a school
4. Community Kitchen program
5. Community Garden
6. Food voucher program (e.g., receiving gift cards for food from a charitable organization)
7. Food delivered by a community program
8. Asking friends or family for help with food
9. Other
10. No – I haven't accessed any food programs

Stress Overall, how well do you think you are coping with stress related to COVID-19 pandemic?
1. Very well
2. Fairly well
3. Not very well

4. Not well at all
5. Don't know
6. Prefer not to say
7. Not applicable – I have not experienced any stress related to COVID-19

Coping 1 Which of the following have helped you to cope with stress related to the COVID-19 pandemic in the past 2 weeks? (Please select all that apply)

1. Connecting with those in my household
2. Connecting with my family or friends virtually (e.g. phone, video chat, etc.)
3. Connecting in-person with friends or family in my "bubble"
4. Enjoying outdoor activities with friends or family
5. Connecting with a mental health worker or counsellor virtually (e.g. via phone, video chat, etc.)
6. Having a supportive employer
7. Spending time with my pet(s)
8. Receiving in-person mental health supports
9. Accessing virtual mental health resources (e.g. online cognitive behavioural therapy, etc.)
10. Maintaining a healthy lifestyle (e.g. balanced diet, enough sleep, exercise, etc.)
11. Keeping up to date with relevant information (e.g. TV news, newspapers, online information, etc.)
12. Limiting my exposure to the news about COVID-19
13. Limiting exposure to social media (e.g. Facebook, Instagram, Snapchat, Twitter etc.)
14. Increasing my use of social media (e.g. Facebook, Instagram, Snapchat, Twitter etc.)
15. Contacting a support group (i.e., where members with the same issues can come together for sharing coping strategies, to feel more empowered and/or for a sense of community)
16. Going for a walk/exercise outside
17. Exercising in my home
18. Doing a hobby
19. Learning or doing something new
20. Volunteering to help
21. Accessing federal government benefits and supports (e.g., Canada Emergency Response Benefit, Canada Emergency Wage Subsidy, Canada Emergency Student Benefit, Employment Insurance, etc.)
22. Accessing provincial government supports (e.g., emergency benefits for workers)
23. Having/anticipating more time for work/domestic duties due to schools re-opening
24. Having/anticipating more time for self-care due to schools re-opening
25. Going to local businesses that are open (e.g., restaurants, hair salons/barber, clothing stores)
26. Other please specify
27. Don't know
28. Nothing has helped me to cope with my stress related to COVID-19
29. Not applicable – I don't feel stressed

Virtual Mental Health 1 If you have experienced mental health challenges at any point during the pandemic, have you used virtual (online or phone-based) mental health services and supports (e.g., counselling, mental health coaching sessions)?

1. Yes
2. No
3. Not applicable. I haven't experienced a mental health challenge during the pandemic

4. Prefer not to say

[If No to Virtual Mental Health 1] Virtual Mental Health 2 Virtual mental health supports are receiving substantial investment to help people cope with mental health challenges during the pandemic. In order to inform better programs, could you please indicate why you did not access virtual mental health supports. (Select all that apply)

1. Stigma
2. Didn't feel I needed help
3. Privacy concerns
4. I don't think they would be helpful
5. I didn't know these supports were available
6. Necessary equipment not available (e.g., computer, smart phone)
7. Connectivity issues (e.g. no/slow internet connection)
8. Competing demands on my time
9. I prefer in-person health care supports
10. Other (Please specify)

Virtual Mental Health 3 Are you aware of any of the following virtual mental health supports in Canada?

1. BounceBack
2. Wellness Together Canada
3. Strongest Families
4. WellCan
5. MindBeacon
6. Other

    1. Yes
    2. No
    3. Don't know /Not applicable/ Prefer not to say

Coping 2 Please indicate how your use of any of the following has been impacted by the COVID-19 pandemic? (Please select one option on each row)

1. Drinking alcohol
2. Use of tobacco products (e.g. cigarettes, cigars, chewing tobacco, vaping, etc.)
3. Use of cannabis products
4. Use of prescribed medication
5. Use of other psychoactive substances (e.g., cocaine, heroin)
6. Gambling
7. Eating/Food consumption
8. Screen time
9. Compulsive online shopping (e.g. buying things you don't really need)

    1. More
    2. Less
    3. No change
    4. Not applicable
    5. Prefer not to say

Coping 3 Has your use of substances increased as a way to cope at any point during the pandemic?

1. Yes
2. No
3. Prefer not to say

SECTION 3: SELF-HARM

The following questions are on the topic of self-harm and suicidal thoughts. We understand this can be a sensitive topic, so please remember that your answers are anonymous. If you are in crisis, please call 1-833-456-4566 toll free (In QC: 1-866-277-3553), 24/7 or visit www.crisisservicescanada.ca

Self-harm 1 Have you done or experienced any of the following, as a result of the COVID-19 pandemic in the past 2 weeks? (Please select one option on each row)

1. Experienced suicidal thoughts/feelings
2. Deliberately hurt myself
3. Worried about someone close to me experiencing suicidal thoughts/feelings or deliberately hurting themselves

    1. Yes
    2. No
    3. Prefer not to say

[If Yes to any of Self-harm 1] Self-harm 2 How often have you done each of the following as a result of the COVID-19 pandemic in the past 2 weeks? (Please select one option on each row)

1. Experienced suicidal thoughts/feelings
2. Deliberately hurt myself
3. Worried about someone close to me experiencing suicidal thoughts/feelings or deliberately hurting themselves

    1. Once a day or more often
    2. Nearly everyday day
    3. A few times a week
    4. Passing thoughts
    5. Don't know
    6. Prefer not to say

SECTION 4: DEMOGRAPHICS

Sex What sex where you assigned at birth?

1. Male
2. Female

Gender We know that gender has important consequences for health and how we are treated by different individuals and institutions. Which gender do you most identify with?

1. Female
2. Male

3. Non-binary
4. Two-Spirit
5. Not listed
6. Prefer not to answer

Ethnicity What is your ethnic origin? Ethnic origin refers to the ethnic or cultural origins of your ancestors (who are usually more distant than a grandparent) (Check all that apply)

1. Indigenous origins (for example, First Nations, Inuit, Métis)
2. East Asian origins (for example, Chinese, Japanese, Korean)
3. South Asian origins (for example, Indian, Punjabi, Pakistani)
4. Southeast Asian origins (for example, Filipino, Thai, Vietnamese)
5. Latin American origins (for example, Brazilian, Cuban, Bolivian)
6. European origins (for example, British, German, Russian)
7. Middle Eastern origins (for example, Iranian, Iraqi, Afghan)
8. African origins (for example, Nigerian, Ghanaian, Zimbabwean)
9. Other (please specify)
10. Don't know
11. Prefer not to answer

Race We know that people of different races do not have significantly different genetics. But our race still has important consequences, including how we are treated by different individuals and institutions. Which race category best describes you? Check all that apply:

1. Black (African, Afro-Caribbean, African Canadian descent)
2. East Asian (Chinese, Korean, Japanese, Taiwanese descent)
3. Southeast Asian (Vietnamese, Cambodian, Thai, Filipino, Indonesian, other Southeast Asian descent)
4. Indigenous (First Nations, Métis, Inuk/Inuit descent)
5. Latino (Latin American, Hispanic descent)
6. Middle Eastern (Arab, Persian, West Asian descent (e.g. Afghan, Egyptian, Iranian, Lebanese, Turkish, Kurdish)
7. South Asian (East Indian, Pakistani, Bangladeshi, Sri Lankan, Indo-Carribean descent)
8. White (European descent)
9. Another race category (Includes values not described above)
10. Do not know
11. Prefer not to answer

Sexuality Do you identify as being LGBT2Q+ (lesbian, gay, bisexual, trans, two-spirit, queer, etc.)?

1. Yes
2. No
3. Unsure
4. Prefer not to answer

Disability Do you identify as a person with a disability?

1. Yes
2. No
3. Prefer not to answer

Pre-Existing Mental Health Do you identify as a person who has a pre-existing (prior to COVID-19) mental health condition?

1. Yes

2. No
3. Prefer not to answer

Citizenship Which of the following best describes your Canadian citizenship status?
1. Canadian citizen by birth
2. Canadian citizen by naturalization
3. Landed immigrant/Permanent resident
4. Refugee
5. Not a citizen
6. Prefer not to say

Children in Household How many children (under 18 years of age) reside in your household?
1. 0
2. 1
3. 2
4. 3+

Parent/Guardian Status Which of the following best describes your parental/guardian status?
1. Not a parent / guardian
2. Parent / guardian (to a child of any age)
3. Prefer not to say

    [If Yes Parent/Guardian] Child Age What age group is/are your child/children? (Please select all that apply)
    1. 4 years and under
    2. 5-11 years
    3. 12-17 years
    4. 18 years and over

Single Parent Are you a single parent?
1. Yes
2. No
3. Prefer not to say

    [If Yes Parent/Guardian] School/Childcare Which of the following best describes the childcare/daycare or school situation of your child/children?
    1. One (or more) of my child/ren is attending childcare/daycare
    2. One (or more) of my child/ren is attending school
    3. One (or more) of my children is homeschooled due to the COVID-19 pandemic
    4. One (or more) of children is homeschooled independent of the COVID-19 pandemic
    5. Other
    6. Prefer not to say

    [If Yes Parent/Guardian] Child Mental Health Compared to before the COVID-19 pandemic and related restrictions in Canada, how would you say the mental health of your child/children is now?
    1. Significantly better now
    2. Slightly better now
    3. About the same
    4. Slightly worse now

5. Significantly worse now
6. It is affecting my children differently (some feel better/some feel worse)
7. Prefer not to answer

[If Yes Parent/Guardian] Child Coping Strategies Which do you think has helped your child(ren) cope with stress related to COVID-19 pandemic in the past 2 weeks? (Please select all that apply)

1. Connecting with family who live outside our home virtually (e.g. phone, video chat, text etc.)
2. Connecting with friends virtually (e.g. phone, video chat, text etc.)
3. Contacting a school or community-based mental health worker or counsellor virtually (e.g. via phone, video chat, etc.)
4. Receiving in-person mental health supports
5. Staying in touch with teachers, school adults, childcare/daycare providers virtually (e.g. phone, video chat, text etc.)
6. Accessing virtual mental health resources through medical professionals (e.g. online cognitive behavioural therapy, etc.)
7. Accessing virtual educational or self-help mental health resources through websites, apps, or phone (e.g., Headspace, KidsHelpPhone)
8. Participating in a virtual child/youth support group
9. Maintaining a healthy lifestyle (e.g. balanced diet, enough sleep, exercise, etc.)
10. Maintaining family routines (e.g., family meals, bedtime routines)
11. Keeping up to date with relevant information (e.g. TV news, newspapers, online information, etc.)
12. Limiting their exposure to the news about COVID-19
13. Limiting their exposure to social media (e.g. Facebook, Instagram, Snapchat, Twitter etc.)
14. More time for social media use (e.g. Facebook, Instagram, Snapchat, Twitter etc.)
15. Going for a walk/exercise outside
16. Exercising in our home
17. Spending time with pet(s)
18. Playing outdoors
19. Playing inside (e.g., games, toys, telling stories)
20. Doing a hobby (e.g., music, reading, arts & crafts)
21. Volunteering to help
22. Connecting in person with friends due to schools/childcare re-opening
23. Connecting in person with teachers/childcare providers due to schools/childcare re-opening
24. Connecting in person with friends outside of school/childcare
25. Other (please specify)
26. Don’t know
27. Not applicable
28. Nothing has helped my child(ren) to cope with stress related to COVID-19

[If Yes Parent/Guardian] Parent-Child Interactions Please indicate how your interactions with your child(ren) have been impacted by the COVID-19 pandemic. (Please select one option on each row)

1. Having quality time with my child(ren)
2. Feeling closeness with my child(ren)
3. Showing love or affection to my child(ren)

4. Observing resilience (strength and perseverance) in my child(ren)
5. Disciplining my child(ren)
6. Conflicts with my child(ren)
7. Using harsh words with my child(ren)
8. Yelling/shouting at my child(ren)
9. Spanking or hitting my child(ren)

    1. More
    2. Less
    3. No change
    4. Not applicable
    5. Prefer not to say

Household How many people reside in your household?

Household Living Arrangements Which of the following best describes your living arrangements? (Please select all that apply)

1. I live alone
2. Living with a spouse or partner
3. Living with friend(s) or housemate(s)
4. Living with siblings
5. Living with my child(ren) who are over 18
6. Living with my child(ren) who are under 18
7. Living with other adult family members (e.g., parents, grandparents)
8. Living with grandchildren
9. Other
10. Prefer not to answer
11. None of the above

Thank you for taking part in this survey. If you've been affected by this topic and would like any more information, need advice, or support, you can go to the following place for help:
Canadian Mental Health Association

Supplementary Table S1. Comparison between parents and the overall rest of the sample (adults without children living at home) reporting mental health outcomes, by survey round.

Round 1 (May 2020)

	**Parents n = 618** [a]		**Overall rest of sample n = 2382** [a]		**Chi Square Test***		
	**n**	**%**	**n**	**%**	**χ2**	**df**	**p**
Parent Worsened Mental Health	**274**	**44.4%**	**847**	**35.6%**	**16.16**	**1**	**<.001**
Not Coping Well	97	16.0%	303	13.2%	3.17	1	0.075
Increased Alcohol Use	**171**	**27.7%**	**383**	**16.1%**	**43.79**	**1**	**<.001**
Suicidal Thoughts/Feelings	**51**	**8.4%**	**125**	**5.3%**	**8.21**	**1**	**0.004**

Round 2 (October 2020)

	**Parents n = 804** [a]		**Overall rest of sample n = 2223** [a]		**Chi Square Test***		
	**n**	**%**	**n**	**%**	**χ2**	**df**	**p**
Parent Worsened Mental Health	327	40.8%	848	38.2%	1.69	1	0.194
Not Coping Well	**154**	**19.6%**	**260**	**12.2%**	**26.01**	**1**	**<.001**
Increased Alcohol Use	**176**	**21.9%**	**328**	**14.8%**	**21.67**	**1**	**<.001**
Suicidal Thoughts/Feelings	66	8.3%	164	7.4%	0.58	1	0.445

Round 3 (January 2021)

	**Parents n = 602** [a]		**Overall rest of sample n = 2432** [a]		**Chi Square Test***		
	**n**	**%**	**n**	**%**	**χ2**	**df**	**p**
Parent Worsened Mental Health	254	42.2%	955	39.3%	1.66	1	0.197
Not Coping Well	**110**	**19.0%**	**324**	**13.8%**	**9.92**	**1**	**0.002**
Increased Alcohol Use	**135**	**22.4%**	**374**	**15.4%**	**17.16**	**1**	**<.001**
Suicidal Thoughts/Feelings	47	7.9%	146	6.1%	2.74	1	0.098

[a] Denominators varied slightly due to missing data.

*Chi square tests compare differences in mental health outcomes between parents and the overall rest of the sample.
